# Recent Weather Extremes and Impacts on Agricultural Production and Vector-Borne Disease Outbreak Patterns

**DOI:** 10.1371/journal.pone.0092538

**Published:** 2014-03-21

**Authors:** Assaf Anyamba, Jennifer L. Small, Seth C. Britch, Compton J. Tucker, Edwin W. Pak, Curt A. Reynolds, James Crutchfield, Kenneth J. Linthicum

**Affiliations:** 1 National Aeronautics and Space Administration, Goddard Space Flight Center, Biospheric Sciences Laboratory, Greenbelt, Maryland, United States of America; 2 United States Department of Agriculture, Agricultural Research Service, Center for Medical, Agricultural, & Veterinary Entomology, Gainesville, Florida, United States of America; 3 United States Department of Agriculture, Foreign Agricultural Service, International Production & Assessment Division, Washington, District of Columbia, United States of America; 4 Universities Space Research Association, Columbia, Maryland, United States of America; 5 Science Systems and Applications Incorporated, Lanham, Maryland, United States of America; The University of Texas Medical Branch, United States of America

## Abstract

We document significant worldwide weather anomalies that affected agriculture and vector-borne disease outbreaks during the 2010–2012 period. We utilized 2000–2012 vegetation index and land surface temperature data from NASA's satellite-based Moderate Resolution Imaging Spectroradiometer (MODIS) to map the magnitude and extent of these anomalies for diverse regions including the continental United States, Russia, East Africa, Southern Africa, and Australia. We demonstrate that shifts in temperature and/or precipitation have significant impacts on vegetation patterns with attendant consequences for agriculture and public health. Weather extremes resulted in excessive rainfall and flooding as well as severe drought, which caused ∼10 to 80% variation in major agricultural commodity production (including wheat, corn, cotton, sorghum) and created exceptional conditions for extensive mosquito-borne disease outbreaks of dengue, Rift Valley fever, Murray Valley encephalitis, and West Nile virus disease. Analysis of MODIS data provided a standardized method for quantifying the extreme weather anomalies observed during this period. Assessments of land surface conditions from satellite-based systems such as MODIS can be a valuable tool in national, regional, and global weather impact determinations.

## Introduction

Severe drought and flooding events occurred in the 2010–2012 period with pronounced impacts on agriculture and public health at regional and national scales. Such extreme weather events frequently result from factors driving interannual climate variability, such as the *El Niño*/Southern Oscillation (ENSO) phenomenon and related tropical and extra-tropical teleconnections [1]–[3]. Importantly, these extremes are increasingly being amplified by long-term shifts in climate and global warming [4], [5]. Extreme weather patterns associated with ENSO have varying impacts on regional ecosystems, agriculture, and health [5]–[10], and these impacts were very evident during the 2010–2012 period. Anomalously wet or dry conditions can lead to ecological settings favouring emergence or re-emergence of disease vectors and pathogens of global public health relevance such as West Nile virus, malaria, dengue virus, cholera, Murray Valley encephalitis virus, and Rift Valley fever virus among others [11]–[16]. Extremes in temperature and rainfall can affect crop yields, crop pests, pasture productivity, and create hazardous fire conditions [17]–[21]. Heavy floods damage infrastructure and crops, and wash away productive topsoil. While socioeconomic impacts of such events vary regionally, depending on existing resources for mitigation, in general they result in increased volatility in agricultural production and commodity prices, as well as higher costs for transport, infrastructure repair, and public health services.

**Figure 1:**
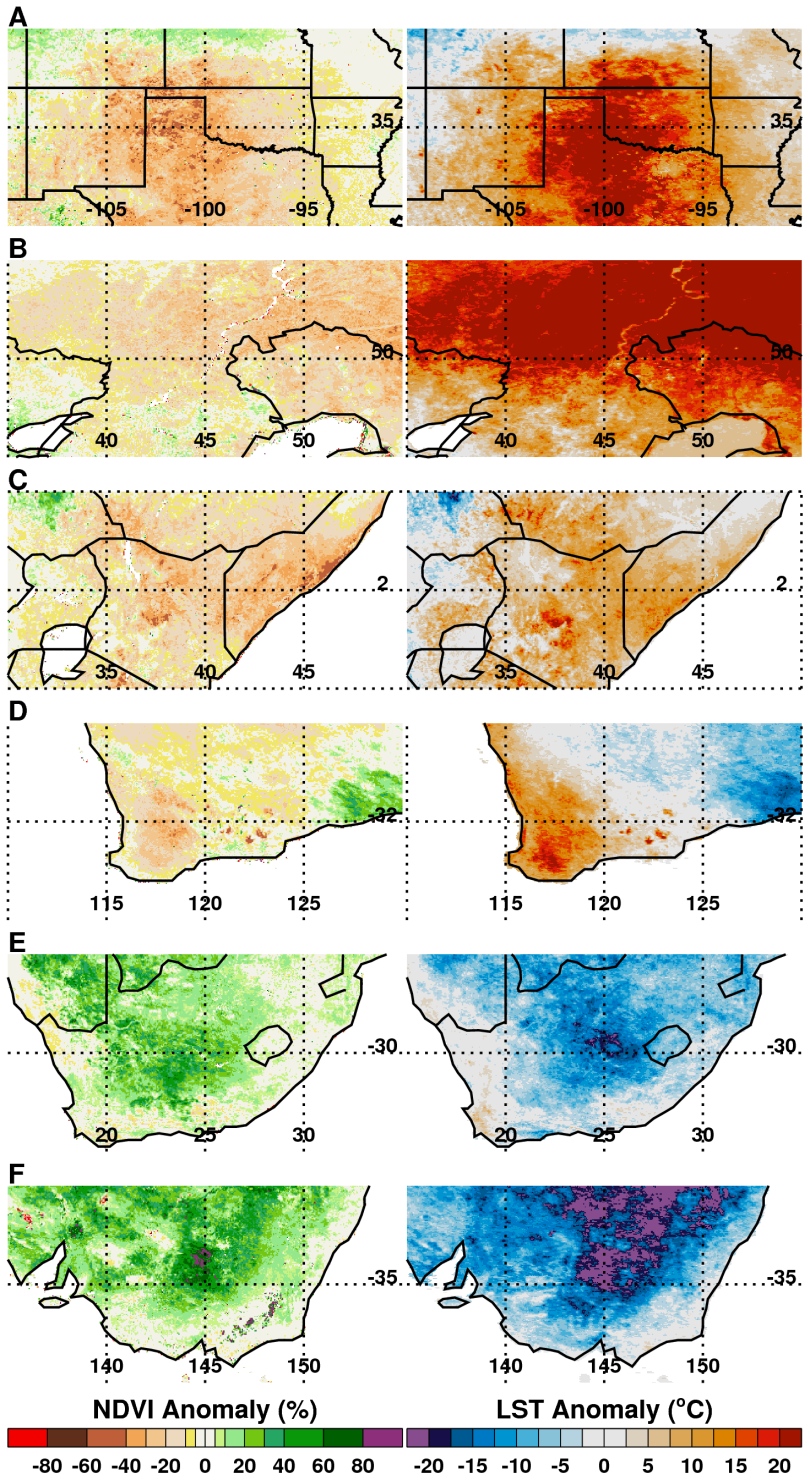
MODIS normalized difference vegetation index (NDVI) and land surface temperature (LST) anomalies for selected growing seasons and regions during 2010–2012. The NDVI quantifies photosynthetic potential of vegetation using satellite-measured reflected radiances in the infrared and red wavelengths [25], [26]. We calculated monthly and seasonal NDVI and LST anomalies by expressing differences between current and mean 2000–2011 NDVI values. NDVI anomalies are expressed as percentage departures, and LST anomalies are absolute departures from long-term means. (A) Texas, US June, July, August (JJA) 2011, (B) SW Russia (Volga District) JJA 2010, (C) East Africa (Somalia/Kenya) DJF 2010/11, (D) SW Australia (Western Australia) SON 2010, (E) South Africa (Free State/North West) DJF 2010/11, (F) SE Australia (New South Wales) SON 2010.

Differences in assessment capability have made it difficult to quantify the global extent and impact of large-scale extreme weather events; however, the 2010–2012 period presents an opportunity to quantify impacts by analysis of satellite-based indicators of surface conditions during these years. We utilized 2000–2012 normalized difference vegetation index (NDVI) and land surface temperature (LST) measurements from the Moderate-Resolution Imaging Spectroradiometer (MODIS) instrument on-board NASA's Earth Observing System Terra and Aqua satellites [22] to illustrate the impacts of weather extremes on agriculture and vector-borne disease outbreak patterns for selected regional locations around the world shown in Figure 1. The anomalous conditions during 2010–2012 are the most extreme weather events in the Terra MODIS 12 year record, and the timing and unique intensity of these events is corroborated by analyses using longer term climate data sets [5], [23]–[24]. We postulate that because both severe drought or flooding may create ecological conditions for the emergence of disease vectors and may substantially affect agricultural production, then under such extreme weather events as documented for 2010–2012 we should observe outbreaks of vector-borne diseases and sharp declines, in the cases of drought, and anomalous increases, in the cases of sufficient above-normal rainfall, in agricultural production. Our aim in this manuscript is to map the locations of documented 2010–2012 extremes at selected regional locations around the world using various satellite datasets, and describe and illustrate the impacts of these extremes by analyzing agricultural production data and vector-borne disease location data for the selected regions.

## Materials and Methods

We analyzed two standard products from the MODIS instrument, the NDVI and LST, in combination with rainfall, crop production data, and vector-borne disease occurrence data extracted from an array of resources to investigate the impacts of weather extremes on agriculture and public health for selected regions of study shown in Figure S1 in File S1. The regions of study were selected based on a number of metrics and observations including assessment of areas of defined geographic anomalies in vegetation index data using the Global Agricultural Monitoring System at http://glam1.gsfc.nasa.gov/ and the Crop Explorer of major agricultural regions at http://www.pecad.fas.usda.gov/cropexplorer/. In addition, we monitored and evaluated vector-borne disease outbreak information from the Program for Monitoring Emerging Diseases (ProMED-mail), World Organization for Animal Health (OIE) (http://www.oie.int/) and US Centres for Disease Control and Prevention data records, available at http://diseasemaps.usgs.gov/wnv_us_human.html and information gathered through collaborations with the Global Emerging Infections Surveillance and Response System (GEIS) division of the DoD Armed Forces Health Surveillance Center (http://www.afhsc.mil/geisPartners). The selected regions and period of study experienced temporally persistent, spatially coherent anomalies and are unique in exemplifying clusters of agricultural production shortfalls and/or increase in vector-borne disease outbreaks associated with background weather anomalies taking place in each region (SI). Where any changes in agricultural production or a vector-borne disease outbreak were associated with extremes resulting from changes in the phase of ENSO, we referred to documentation from the NOAA Climate Prediction Center for information. Details of the data sets and analyses are given in the SI Materials and Methods (File S1), including Table S1 and Figures S1 to S4 in File S1. The NDVI quantifies the photosynthetic capacity of vegetation and how this varies in time [25], [26], and LST provides a measure of the temperature of the land surface [27]. Both data sets can be used to monitor and assess agricultural growing conditions [28] and also infer ecological conditions that lead to the emergence and increase in abundance of disease vectors [16]. To gauge the impact of extreme weather events on regional ecosystems, we calculated monthly NDVI and LST anomalies by expressing differences between the monthly values and long-term mean 2000–2011 NDVI or LST values [29]. We also derived seasonal anomaly metrics by calculating cumulative three month anomalies, for example June, July, August (JJA) [SI]. The LST and NDVI monthly and seasonal anomalies were examined over the growing seasons for the selected six global agricultural regions worldwide (Figure 1 and Figure S1 in File S1) and were contrasted with agricultural production data extracted from the USDA Production, Supply, and Distribution Online (PSD) electronic database (http://www.fas.usda.gov/psdonline/) for the focal regions. Seasonal NDVI is particularly relevant to agriculture, as cumulative NDVI is linearly related to gross primary production, and hence highly correlated to agricultural production [30]. Accordingly temperature (LST) during the growing season regulates crop growth [7], [18], [19] and in general vegetation, which in this case can be observed through NDVI time series. Anomaly calculations using MODIS data provided unbiased and spatially continuous representations of the impacts of extreme weather conditions on regional vegetation for the study period and were augmented with rainfall data extracted and derived for the focal regions from the Global Precipitation Climatology Project [31] (Table 1). Various publicly-available databases were surveyed to develop maps of outbreak locations of dengue, Rift Valley fever, Murray Valley encephalitis, and West Nile virus disease (SI), and these maps were juxtaposed with the focal anomalous regions identified in this study. We also compared the seasonal distribution of LST and NDVI values for the focal regions in the study period to gauge the direction of shifts of the variables from long-term means resulting from extreme conditions. Historical ENSO patterns and timing were determined from NOAA data portals (http://www.cpc.ncep.noaa.gov/products/MD-index.shtml) to establish that extreme weather conditions associated with the cold *La Niña* phase of ENSO and associated teleconnections resulted either in severely depressed vegetation conditions/drought and above-average LST, or excessive rainfall, denser than average vegetation, and cooler than normal LST, with a heterogeneity of impacts on agricultural production and vector-borne disease transmission in the various focal regions around the world.

**Table 1:** Total seasonal rainfall, long-term means, and anomalies for 2010–2011 extracted from the Global Precipitation Climatology Project [31] for regions presented in Figure 1.

Region	Season	Total (mm)	Mean (mm)	Anomaly (%)
US (Texas)	June–August 2011	59.40	174.11	−65.88
SW Russia (Volga District)	June–August 2010	35.48	133.18	−73.36
East Africa (Somalia/Kenya)	December 2010–February 2011	7.68	51.86	−85.19
SW Australia (Western Australia)	September–November 2010	25.84	90.08	−71.31
South Africa (Free State/North West)	December 2010–February 2011	363.92	253.69	43.45
SE Australia (New South Wales)	September–November 2010	255.27	102.93	148.00

Anomalies are calculated based on the 1979–2011 climatology period.

### Ethics Statement

All disease case data analyzed were anonymized. We used GPS latitude-longitude coordinates to map approximate case locations, and we did not handle or deal with any human or animal specimens.

## Results

### Regions Affected by Drought: Below-Normal Rainfall and Above-Normal Temperatures

**Figure 2:**
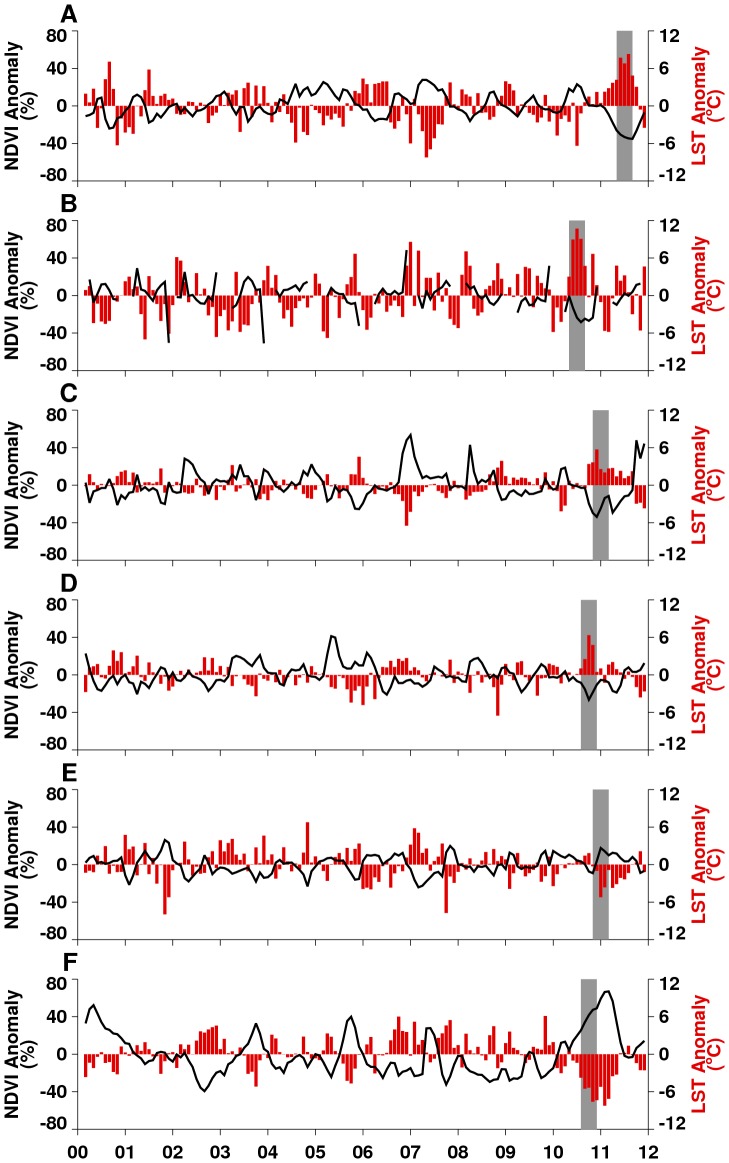
Monthly time series of 2000–2012 MODIS normalized difference vegetation index (NDVI; black line) and land surface temperature (LST; vertical red bars) anomalies for agricultural regions shown in Figure 1 and Figure S1 in File S1. Grey bars indicate selected periods of focused impact studies for each region.

**Figure 3:**
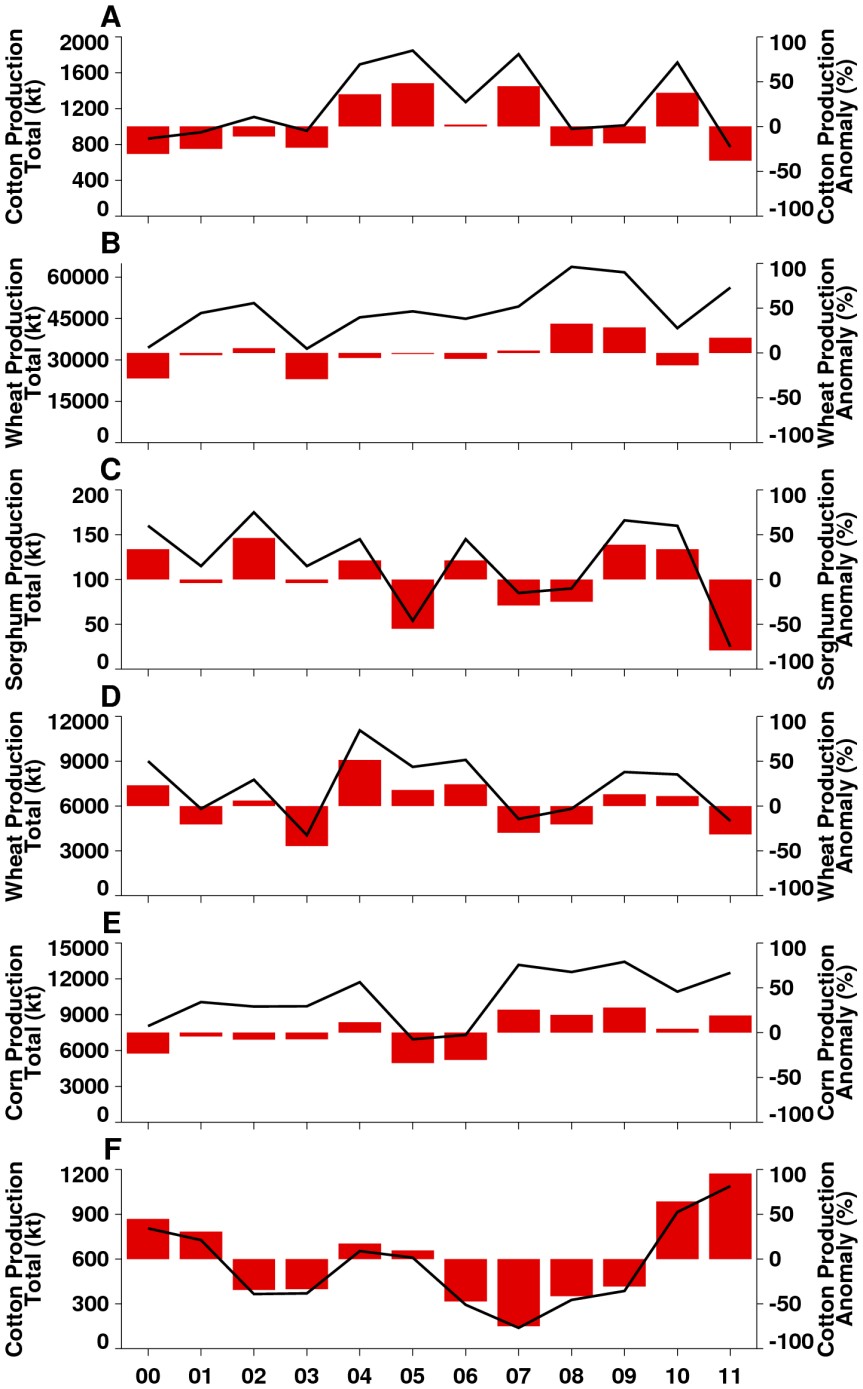
Crop production time series for selected regions in 2000–2011 expressed as annual totals (represented by the black line) and as annual anomalies, i.e., departures from the decadal mean (represented by the red bar chart).

**Figure 4:**
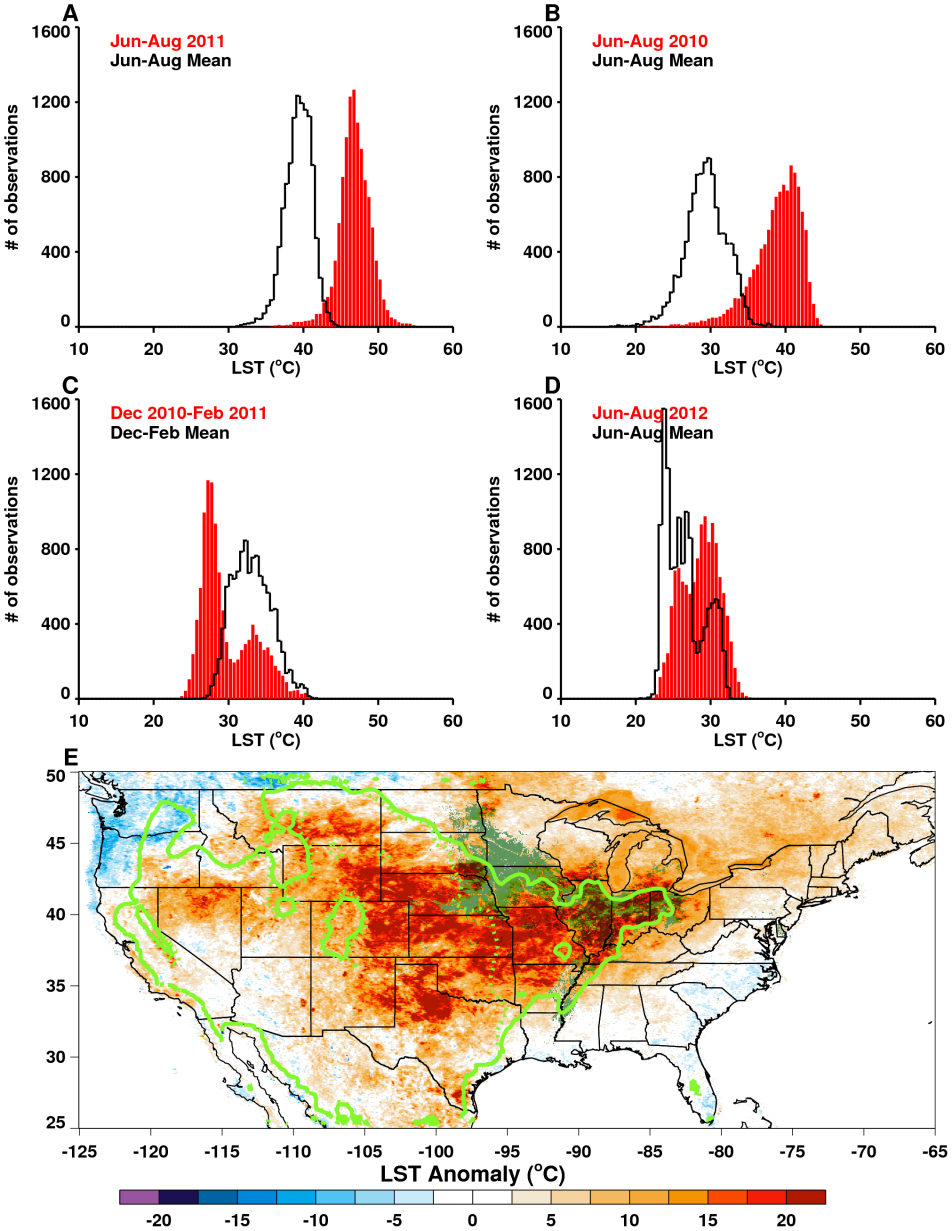
(A–D) Histograms of MODIS growing season land surface temperature (LST) distributions (red bars) compared against the long-term means (black lines) for selected regions of study. (E) Spatial pattern of LST anomalies for the US during June, July, August (JJA) 2012. The area shaded in olive green shows the dominant corn and soy growing region; the solid neon green line delineates the JJA 2012 seasonal 30°C isocline which encompasses the majority of the US agricultural region; and the dotted neon green line shows the JJA 2000–2011 long-term mean 30°C isocline. Persistent temperatures above 30°C destroy most crops and reduce yields [6], [17], [57].

**Figure 5:**
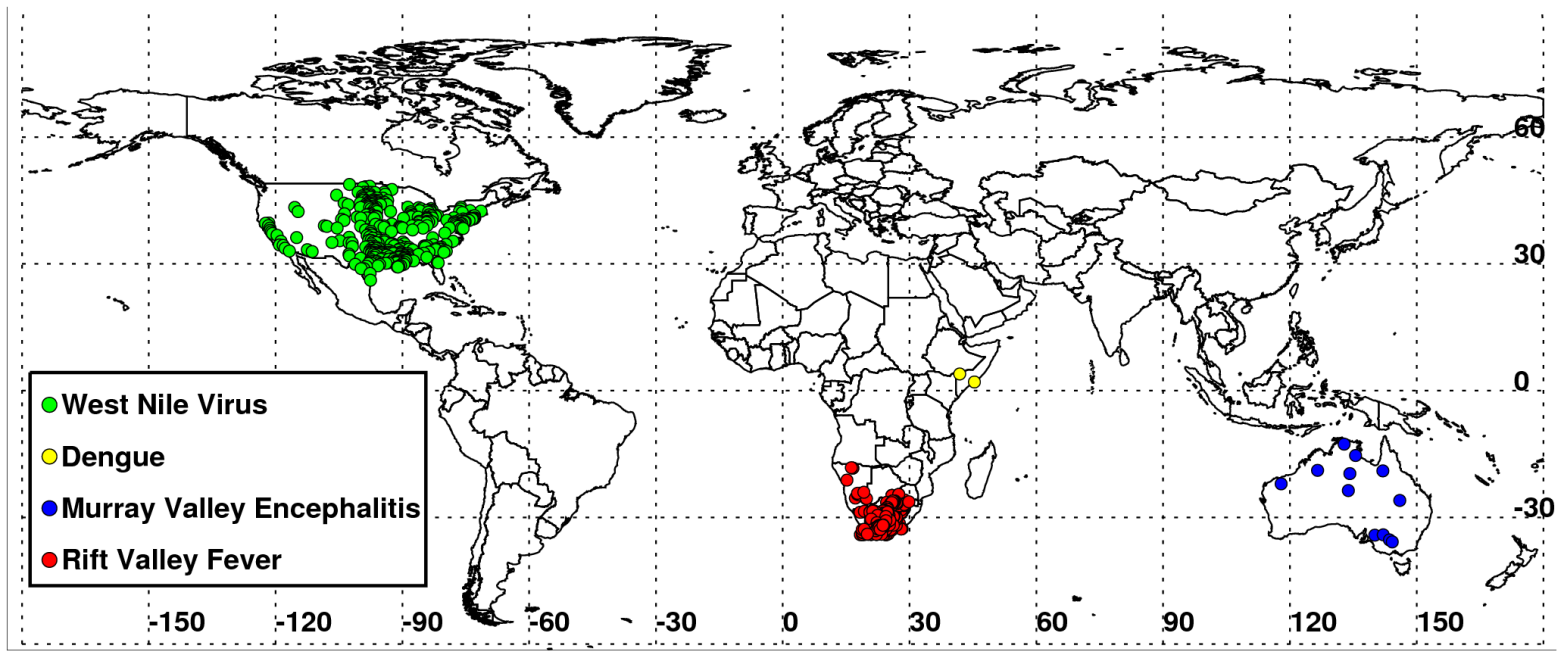
Global distribution of epidemics/epizootics of vector-borne disease outbreaks during 2010–2012 associated with weather extremes, showing the outbreak locations of West Nile virus disease (US, 2012), dengue (East Africa, 2011), Rift Valley fever (Southern Africa, 2011), and Murray Valley encephalitis (Australia, 2011).

In 2010 and 2011, severe drought affected four regions: the southern United States, western Russia, Western Australia, and East Africa (Figure 1A–D); in 2012, the entire continental US was affected (Figure S2 in File S1). In all cases, drought reduced crop yields and led to increased wild fires. The southern half of the contiguous US experienced extreme and persistent drought from mid-2010 through September 2011, with the greatest impact in Texas (Figure 1A). The Texas drought epicenter had historic rainfall lows of up to ∼66% below normal (Table 1), coupled with one of the hottest summers on record in 2011 [5], [24], [32], with monthly LSTs up to 8°C above normal (Figure 2A), and seasonal LST as high as 20°C above normal (Figure 1A). As a result of high temperatures, vegetation photosynthetic capacity was reduced by 40–60% below average (Figures 1A and 2A). Drought and scorching temperatures led to declines in crop conditions and abandonment of fields. In particular, this was because the Texas drought of 2011 was of historical proportions and classified as the most extreme rainfall deficit year in over 100 years [24], [32]. The severity of the drought overwhelmed the agricultural system with high evapo-transpiration rates, rationing of ground water resources for irrigation and other uses. Although producers switched irrigation from corn to more drought-tolerant cotton in some areas, cotton production still declined by 50% to 771 kilotons below the 2000–2011 average (Figure 3A). Extreme water deficits had reduced the capacity of the cotton crop to carry fruit as a result of lower rates of leaf photosynthesis and severely reduced the development of floral buds thus reducing yields [33]. Direct losses from the drought approached $10 billion in the state of Texas alone in 2011 [34], [35]. Hot, dry conditions also resulted in a very active fire season across the southern US costing more than $1 billion [36]. Unprecedented 100-year climate conditions of extreme high temperatures (Figure 4A,E) and lack of rainfall were linked to the highest period of West Nile virus activity on record in Texas and the rest of the continental US: the 2012 epidemic of West Nile virus disease across the continental US (Figure 5) was the largest such outbreak since the introduction of West Nile virus into the country in 1999, and the spike in human West Nile virus disease cases in 2012 can in part be associated with extreme drought [12] and anomalously high positive shift in summer mean temperatures from ∼30°C to 33°C (Figure 4A). Elevated temperatures increase the efficiency of transmission of West Nile virus by both *Culex pipiens* and *Cx. tarsalis* mosquitoes, and have positive effects on mosquito population development and survival, biting rates, and viral replication within these mosquito species [37]–[39].

Across Eurasia, the summer drought of June-August 2010 was centered in western Russia (Figure 1B) with the drought area extending to Belarus, Poland, Germany, Ukraine, and Kazakhstan [23], [40]. Cumulative seasonal LSTs reached as high as 20°C above normal with declines in NDVI of up to 40% below normal (Figures 1B and 2B). Severe and persistent drought sharply reduced agricultural production of major grains in Russia's Central, Volga, and Ural Districts. Estimated wheat production for 2010 was just over 41,500 kilotons, approximately 14% below the 2000–2011 average (Figure 3B), and barley production for 2010 was estimated at 8,350 kilotons, which was 51% below the 2000–2011 average and the lowest in that time span. The combination of severe drought and high temperatures led to the abandonment of approximately 13.3 million hectares of cropland, including half the grain area in the Volga District. The extreme heat resulted in more than 15,000 deaths in Russia during the 2010 summer [23], [40]. As in the southern US, the drought caused extreme fire conditions. From July-September 2010 more than 1.25 million hectares burned, causing an estimated $630 million in damages [41].

East Africa experienced below-normal rainfall from early 2010 through mid-2011. Rainfall for December 2010 – February 2011 was ∼85% below normal (Table 1) and seasonal LSTs were between 5–20°C above normal. The stress of these conditions on vegetation and pastures is illustrated by NDVI departures of 60% below normal (Figure 1C). The peak of the East Africa drought was during the 2010–2011 *La Niña* period (Figure 2C) and led to sharp declines in agricultural production, in particular sorghum, a staple food in this semi-arid region. For Somalia, the total sorghum production for 2011 was 25 kilotons, more than 80% below normal and the lowest for the last decade (Figure 3C). The drought diminished the productivity of pastures and caused widespread famine and high mortality in livestock throughout the region. Although it is difficult to gauge the total human impact of the East African famine, the United Nations estimated more than 13 million people required humanitarian assistance there by December 2011 [42]. Persistent above-normal temperatures were associated with the first known large-scale outbreak of dengue in East Africa. Dengue is a mosquito-borne disease associated with infrequent replenishment of stored water supply around households during hot, dry climatic conditions in densely populated areas [9], [15], [43], which has been shown to increase populations of *Aedes aegypti*, the primary mosquito vector of dengue [44]. Severe drought coupled with higher temperatures increased the abundance of container-breeding dengue virus vector mosquitoes in urban settings leading to the dengue outbreak that persists up to the present [45]. Outbreaks were centered in Mogadishu, Somalia, and Mandera, Kenya (Figure 5), compounding the effects of famine on already vulnerable populations.

Australia experienced both extremes: drought over the western quarter and extreme wet conditions over the eastern half of the continent. Drought and floods in Australia are linked with ENSO cycles, as seen in 1916, 1917, 1950, 1954–1956, 1973–1975, and 2010–2011 [46]. In western Australia, drought prevailed during the 2010 growing season: seasonal LSTs were up to 20°C above normal, vegetation conditions were 30–60% below normal (Figures 1D and 2D), and total rainfall for September – November 2010 was only ∼26 mm, or ∼71% below normal (Table 1). Regional wheat production was more than 30% below normal at just under 5,000 kilotons (Figure 3D). The persistent drought, low soil moisture, and above-normal temperatures centered on Western Australia's central agricultural region resulted in the worst overall agricultural production in 40 years [47].

### Regions Affected by Excessive Rainfall and Below-Normal Temperatures

Three regions experienced excessive rain, floods, and below-normal temperatures during the September 2010 to May 2011 *La Niña* event [48]: the northern US and southern Canada; southeastern Australia; and southern Africa. In North America, heavy spring rains saturated fields, causing flooding and hampering field work in portions of the Corn Belt, Ohio Valley, Tennessee Valley, Missouri Valley, Pacific Northwest, and the northern Great Plains extending into southern Canada. The northern and central Great Plains and much of the Northeast and Atlantic Coast States accumulated rainfall totaling 200% or more above normal and experienced prolonged cool temperatures during this period (Figure S3 in File S1). Above-average precipitation also fell in the Pacific Northwest and rains triggered widespread flooding in the Mississippi and Missouri basins, putting thousands of acres of farmland under water and causing more than $5 billion in damage. Agricultural losses in southern Canada amounted to $1 billion, but impacts on US agricultural production were mixed: although states like Iowa reported bumper harvests, most agricultural states suffered reduced harvests from delayed planting of crops in spring 2011 because waterlogged fields were inaccessible [48], [49].

The main corn producing region of South Africa, known as “The Maize Triangle” received heavy rainfall during the 2010–2011 *La Niña* [48], with December 2010-February 2011 total rainfall more than 360 mm, or ∼43% higher than normal (Table 1). Abundant rainfall combined with cooler temperatures (Figures 1E and 2E) created favorable growing conditions throughout the country and resulted in the fifth successive year of 25% above-average maize production (Figure 3E). Cool, wet weather led to rapid green-up of vegetation (Figure 1E) and flooding of low-lying areas, or *dambos*/*pans*, creating ideal ecological conditions for hatching *Aedes* mosquito eggs infected with the Rift Valley fever virus. These conditions in association with the downward shifts in December-February 2010/2011 mean seasonal temperatures from ∼40°C to 30°C in South Africa (Figure S4C in File S1) were conducive not only to increased mosquito populations, but increased virus infection in mosquitoes [50], [51] and subsequent Rift Valley fever virus transmission. This resulted in the most extensive and widespread outbreak of Rift Valley fever in the region since the 1970s (Figure 5), negatively impacting the livestock industry and human health in southern Africa [51], [52]. Overall, MODIS time series NDVI and LST anomalies during the 2010/11 *La Niña* were the most persistent and extreme anomalies for the southern Africa region for the 12 year record.

Southeastern Australia likewise experienced extreme wet conditions, with September-November 2010 rainfall total of ∼255 mm, or 148% above normal (Table 1). High rainfall and cooler temperatures (Figure 1F) resulted in increased agricultural yields, putting Australia on track for one of the largest cotton crops in the last decade. The USDA estimated 2011 total cotton production in the region at 1,088 kilotons, approximately 95% higher than the 2000–2011 average (Figure 3F). Most cotton growing regions in New South Wales and Queensland received above-normal winter and spring rainfall, creating excellent planting conditions and sufficient irrigation resources. Overall, 2011 area planted and production estimates were at decadal highs and represent a significant recovery from severe droughts experienced during 2006–2009 (Figure 2F). However, the wet conditions also had negative impacts. The large accumulation of biomass in 2011 created heavy fuel loads that resulted in an intense fire season in early 2012, with large brush fires occurring in Victoria, south Australia, and New South Wales [53], [54]. Rains also caused moderate to severe flooding in parts of eastern Australia, especially central-southern Queensland and northern New South Wales. At the peak of the *La Niña* event from November 2010 to January 2011, floods damaged crops in low-lying areas. The combined persistent and heavy rainfall, cooler temperatures, and vegetation growth created ideal conditions for an increase in populations of mosquito vectors of Murray Valley encephalitis virus, leading to outbreaks over northern and eastern Australia [55] (Figure 5). Murray Valley encephalitis mosquito vectors, primarily *Culex annulirostris*, favor cooler temperatures associated with heavy rainfall periods in the tropics and sub-tropics [56]. The downward shift from ∼40°C to 30°C in December-January 2010/2011 mean seasonal temperatures compared to the long-term mean distribution for eastern Australia (Figure S4E in File S1) during this epidemic period shows that a cooler environment was conducive to increased mosquito populations and virus amplification, and increased virus transmission to humans.

## Discussion

The above regional snapshots exemplify the contrasting patterns and impacts of extreme weather events and demonstrate that changing weather will vary geographically in both magnitude and extent. For example, Figure 4 shows seasonal LSTs against long-term mean seasonal LST distributions for selected regions during the study period. June-August LST distributions for both Texas, US (2011, Figure 4A) and Volga District, Russia (2010, Figure 4B) show significantly warmer and drier conditions compared to long term June-August means, with temperatures as high as ∼45°C. Importantly, seasonal minimum temperatures for both regions were also ∼10°C higher than normal, indicating an overall increase in seasonal LST, not simply increased LST variability. Temperatures above 30°C increase stress on vegetation and damage pastures, a variety of crops [6], [7], [17]–[20], [57], and here led to sharp declines in agricultural production, such as a 50% decline in cotton production in Texas (Figure 3A) and a 25% decline in Volga District wheat (Figure 3B). In contrast, Figure 4C shows seasonal LSTs for December 2010-February 2011 over South Africa's Maize Triangle. Compared to Texas and Volga District, the weather in South Africa was cooler and wetter, with average temperatures in the range of 19–28°C that have been associated with increased corn yields [7], [18], [19], [57]. Indeed, above-average corn production was observed over South Africa during the last five years (Figure 3E).

The 2011 drought effects in Texas were similar to the Iowa corn-growing area a year later (Figure 4D), where the 2012 seasonal minimum, mean, and maximum temperatures as high as 35°C, were all above normal, with higher monthly and long-term mean LSTs. The 2011–2012 US droughts were associated with negative public health impacts in addition to large scale agricultural impacts such as reduction in production of major commodities including corn and cotton. The spatial pattern in Figure 4E shows that in 2012 the drought epicenter had shifted to the central US unlike in 2011 when the drought was centered in Texas (Figure 1A). This shows the tendency for drought to persist, expand, and propagate over large areas once initiated. Extreme 2012 summer temperatures affected ∼70% of the US corn growing area and led to extensive impoverishment of pasture and rangeland, plus record reductions in corn production. The widespread and persistent nature of drought and high temperatures also contributed to the large-scale epidemic of West Nile virus disease cases across the continental US [38] (Figure 5; SI).

Droughts of the magnitude we observed in the US, Russia, and southeastern Australia have produced recent volatility in agricultural commodity prices, increased costs for feed and water in the livestock industry, decreased supply of water resources, increased fire risk, and contributed to other detrimental societal impacts [17]. The 2012 growing season marked the third consecutive year of weather extremes that affected the northern hemisphere and resulted in depleted commodity stocks. The major 2010–2012 droughts were the most persistent and extreme for these regions in the 2000–2012 MODIS record. The 2011 drought in eastern Africa was also the most extreme event in the MODIS record for the region. Conversely, *La Niña* rains in Australia and South Africa had largely positive impacts on agriculture, but associated vector-borne disease outbreaks negatively impacted public health. Our findings show that extreme seasonal shifts in weather conditions, regardless of direction, favor different vector-borne disease systems in different ways and may lead to increased risk of vector-borne disease outbreaks. Transmission of different vector-borne pathogens such as West Nile virus or Rift Valley fever virus may be enhanced by opposite weather extremes because they are different viruses with completely different ecologies of transmission, different hosts, different mosquito vectors, and different optimal habitats. For instance, anomalously hot and dry conditions can lead to increased storage of water around households and consequent increases in populations of container-breeding mosquitoes like *Aedes aegypti* that may transmit dengue virus. On the other hand, anomalously prolonged wet conditions and flooding, aside from triggering the large-scale emergence of disease-vector mosquitoes, can enhance production and sustainment of vegetation which directly favors the survival of mosquitoes and thereby their vectorial capacity, that is, their capacity to become infected by viruses, such as Rift Valley fever virus, and then transmit them. Such environmentally enhanced outbreaks will vary globally depending on the virus and its transmission ecology and the geographic location and baseline condition of disease endemism and seasonality [58], and could favor the globalization of such pathogens. Our analysis of temperature and vegetation conditions provides a method for quantifying weather extremes consistently from region to region, and demonstrates the value of satellite data in monitoring and mapping the magnitude and extent of such events. As extreme weather events become more common under a more variable climate, nations will face costly adaptation. Systematically-collected satellite data such as those we describe here can be a valuable contribution to national, regional, and global weather impact determinations.

## Supporting Information

File S1 Supplementary Information. This SI section includes: Detailed Materials and Methods Text, Figures S1 to S4, Table S1, References, Figure Legends.
(DOCX)
