# Supplementary material for: Recent Weather Extremes and Impacts on Agricultural Production and Vector-Borne Disease Outbreak Patterns
Source: PLoS One. 2014 Mar 21;9(3):e92538. doi: 10.1371/journal.pone.0092538 (PMC3962414; doi:10.1371/journal.pone.0092538)
Supplement: File S1 — Supplementary Information. This SI section includes: Detailed Materials and Methods Text, Figures S1 to S4, Table S1, References, Figure Legends. (DOCX) [file pone.0092538.s001.docx]

**Supporting Information (SI)**

**Recent weather extremes and impacts on agricultural production and vector-borne disease outbreak patterns**

Assaf Anyamba^1*^, Jennifer L. Small^1^, Seth C. Britch^2^, Compton J. Tucker^1^, Edwin W. Pak^1^, Curt A. Reynolds^1^, James Crutchfield^3^, Kenneth J. Linthicum^2^

National Aeronautics and Space Administration-Goddard Space Flight Center, Biospheric Sciences Laboratory, Greenbelt, Maryland, United States of America

^2^ United States Department of Agriculture-Agricultural Research Service, Center for Medical, Agricultural, & Veterinary Entomology, Gainesville, Florida, United States of America.

3 United States Department of Agriculture-Foreign Agricultural Service, International Production & Assessment Division, Washington, District of Columbia, United States of America

^&^ Universities Space Research Association, Columbia, Maryland, United States of America

^#^ Science Systems and Applications Incorporated, Lanham, Maryland, United States of America.

*Email: assaf.anyamba@nasa.gov

**This SI section includes:**

Extended Materials and Methods

**Figures S1** to **S4**

**Table S1**

References

**Materials and Methods**

**NDVI and LST Data Sets**

Both the normalized difference vegetation index (NDVI) and land surface temperature (LST) data sets used in this study were derived from NASA’s Earth Observing System Moderate Resolution Imaging Spectroradiometer (MODIS) instrument aboard the Terra (EOS AM-1) spacecraft. Terra has a sun-synchronous, near-polar circular orbit with an equatorial crossing time of approximately 10:30 am in a descending node. MODIS has consistently acquired data in 36 discrete spectral bands since the launch of Terra in late February 2000. The 2,330 km viewing swath of the MODIS instrument allows a look at every point on the globe within a 1-2 day repeat cycle. MODIS improves upon the heritage NOAA Advanced Very High Resolution Radiometer (AVHRR) instrument series with finer spatial resolution, shorter repeat cycle, and smaller spectral bandwidths. We used the MODIS global monthly Climate Modeling Grid (CMG) products with a spatial resolution of 0.05° latitude and longitude (~5.5 km). The CMG products are generated from the full resolution (250 m to 1000 m) MODIS data in order to facilitate global scale analyses such as described in this study. We used MODIS NDVI and LST products jointly to infer aggregate ecosystem conditions over global agricultural areas and in regions affected by vector-borne disease outbreaks from June 2010 to August 2012. We describe the NDVI and LST in more detail below; further information on all MODIS land products can be found at https://lpdaac.usgs.gov/products/modis_products_table.

The NDVI and similar vegetation indices are widely used to infer the photosynthetic capacity of vegetation and are used as a land surface input in various weather, climate, biogeochemical, and hydrological models [1]. Applications of NDVI are numerous and varied and include agricultural monitoring, famine early warning, and determination of land use and land cover changes, among others. The NDVI is simply the ratio of the difference between the near-infrared and red reflectance to their sum; since green leaves with dense chlorophyll are more reflective in the near-infrared wavelengths than in the visible, this ratio is higher (approaching one) for healthy green vegetation and lower (approaching zero) for stressed vegetation [2]. MODIS NDVI data are derived from the red and near-infrared bands, centered at 648 nm and 848 nm, respectively. The band reflectance data are atmospherically corrected and masked for cloud, cloud shadow, and aerosol contamination [3]. In this study we used the global monthly 0.05° MOD13C2 NDVI; this product has been aggregated from 250 m MODIS NDVI as described above.

Land surface temperature (LST) is a key parameter in land surface processes. The difference between LST and surface air temperature drives energy exchange at the planetary boundary layer. Changes in LST can induce convection at the boundary layer and influence air temperature, surface winds, cloudiness, and precipitation. LST is used in global climate models for heat budget and boundary layer energy balance calculations, and has also proved useful for agricultural applications in estimating crop water demands and drought severity assessments [4]. When plants encounter water deficit, transpiration is reduced causing leaf temperature (which is equal to LST for dense vegetation) to rise. On the other hand, when water is not limited, transpiration will result in cooling of the leaf temperature relative to air temperature. We used LST to estimate plant water availability where vegetation is dense, such as in agricultural areas during the growing season. In this study we used the global monthly 0.05° MOD11C3 data set. MODIS LST is derived from daytime and nighttime thermal infrared measurements in bands 31 (10.8-11.3 nm) and 32 (11.8-12.3 nm) using the day/night LST algorithm described in [5]. Cloud screening is performed using the MODIS cloud mask product (MOD35_L2) prior to the LST calculation. Estimated accuracy for the MODIS LST product is 1 degree Kelvin for land cover types with known emissivity.

For this study we were interested in weather extremes and their impact on crop production in large agricultural regions and how these extremes contributed to vector-borne disease outbreaks. Since crop production is influenced over an entire season rather than a single month, we first aggregated the NDVI and LST data from monthly to three-monthly to better represent a typical growing season. We calculated NDVI and LST sums for each three-month growing season for each year from the start of the Terra MODIS record in March 2000 through December 2012:

Here, m=1, 2, 3 represents the three months in growing season *s* and the year *y*. We then calculated the average sum for each season, taken over all years from 2000 to 2012

Lastly, we calculated seasonal NDVI and LST anomalies for all seasonal periods from June 2010 to August 2012. Normalizing by the mean isolates areas with high variability in the vegetation (temperature) signal and establishes a meaningful historical context for the current NDVI (LST) to determine relative severity [6]. The seasonal anomalies measure the departure of the given season from the average season, and are defined as:

$$\text{LST\_ANOM}_{\text{s,y}} =\text{LST}_{\text{s,y}} -\overline{\text{LST}_{\text{s}} }$$

The NDVI anomaly is expressed as a percentage of the average due to the non-intuitive nature of NDVI units. The LST anomaly is expressed in absolute units of temperature (degrees Celsius).

The resulting growing seasonal anomalies were mapped and areas with large departures from mean values for high density of agricultural land identified. Regional NDVI and LST anomaly maps for each season and region of interest are presented in **Figure 1**. We identified agricultural regions using a set of satellite-based crop classifications; the data sources varied by region and are presented in **Table S1**. We selected seven regions for further analysis. For each region, we extracted all monthly NDVI and LST values from a 3°x3° area of highest agricultural density as defined by the crop classification data. The crop classification and the selected areas are presented in **Figure S1**.

We created monthly time series of LST and NDVI anomalies by averaging all valid 0.05° grid cell values within the 3°x3° box for each month and year; the resulting time series are presented in **Figure 2**. The time series plots represent the variability in LST and NDVI over the MODIS Terra record for each agricultural region and allow comparison of the 2010-2012 seasonal LST and NDVI with those in prior growing seasons.

In order to compare LST and NDVI values for 2010-2012 extreme anomaly regions with those in an average season, we plotted all 0.05° grid cell values for selected regional 2010-2012 growing seasons along with the grid cell values for the average 2000-2012 season as a histogram. These results are presented in **Figure 4** and **Figure S4**.

**ENSO Data**

We surveyed the NOAA online repository of historical ENSO indices, found at http://www.cpc.ncep.noaa.gov/products/MD-index.shtml, to cross-reference the timings and locations of anomalous weather patterns, and related downstream agricultural and public health impacts, inferred from MODIS NDVI and LST data, with historical timings of global *La Niña* and *El Niño* events.

**Agricultural Production Data**

We obtained crop production data for all regions outside the US from the USDA Foreign Agricultural Service (FAS) *Production, Supply, and Distribution* (PSD) online database. This database contains current and historical official USDA data on production, supply, and distribution of agricultural commodities for the United States and key producing and consuming countries. The database can be accessed at http://www.fas.usda.gov/psdonline/. For the US regions, production data were sourced from the USDA National Agricultural Statistics Service database located at <http://quickstats.nass.usda.gov/>. For each region, we obtained production data for the dominant crop type in the classification from 2000-2011 (**Table S1**) to match the MODIS time series record. Where crop type was not available in the crop classification we used data from the USDA FAS *Crop Explorer Crop Area Maps,* available at http://www.pecad.fas.usda.gov/cropexplorer/, to determine the dominant crop. We converted all production data to common units of metric kilotons. Cotton production totals for Texas and southeastern Australia were given in number of 480 pound bales; these totals were converted to kilotons with multiplication by (480*0.453592*10^-6^). All other production totals were given in metric tons and were converted to kilotons with multiplication factor (10^-3^). We then calculated average 2000-2011 production totals, annual production anomalies, and finally annual percent anomalies (pct_anom) for each year *y* as:

Crop production totals and anomalies for each year and region are presented in **Figure 3**.

**Rainfall Data**

Lower than normal NDVI (negative NDVI anomaly) and higher than normal LST (positive LST anomaly) are in general associated with below-normal crop production. Conversely, higher than normal NDVI and lower than normal LST are associated with above-normal crop production. This relationship exists because higher NDVI and lower LST are both associated with adequate rainfall that benefits crop productivity [7,8]. To illustrate the strong relationships among LST, NDVI, and rainfall we used the Global Precipitation Climatology Centre (GPCC) *Global 1° Monitoring Product*, available at:

ftp://ftp-anon.dwd.de/pub/data/gpcc/html/ monitoring_download.html, to compare rainfall totals for each region and season of interest to the seasonal means. We aggregated the monthly GPCC data to seasonal sums, calculated the 2000-2011 average seasonal sums, and finally the seasonal rainfall percent anomalies for each season of interest. Seasonal rainfall totals, means, and percent anomalies are presented by region in **Table 1**.

**Vector-borne Disease Outbreak Data**

Unprecedented mosquito-borne disease outbreaks in East Africa (dengue), South Africa (Rift Valley fever), Australia (Murray Valley encephalitis), and the United States (West Nile fever) occurred during our period of study. This indicates how extreme weather events influenced the emergence and propagation of mosquito vectors that lead to disease outbreaks [9,10]. We identified locations of dengue outbreaks (East Africa) and Murray Valley encephalitis activity (Australia) by searching online reports of *The Program for Monitoring Emerging Diseases* [ProMED] (http://promedmail.com/pls/apex/f?p=2400:1000). This global database provides early warning reports of emerging disease outbreaks worldwide. Map coordinates for each named outbreak location were approximated using online sources such as the *Latitude/Longitude Finder* (http://www.satsig.net/maps/lat-long-finder.htm). Locations of Rift Valley fever outbreaks in Southern Africa were obtained from the online data records of the World Organization for Animal Health (OIE) (http://www.oie.int/). County-level locations of human West Nile fever cases were extracted from US Centres for Disease Control and Prevention data records, available at http://diseasemaps.usgs.gov/wnv_us_human.html and http://www.cdc.gov/ncidod/dvbid/westnile/Mapsincidence/surv&control12IncidbyCounty.htm. We mapped all location data as shown in **Figure 5**.

Outbreaks of Rift Valley fever and Murray Valley encephalitis followed periods of sustained above-normal rainfall in southern Africa and southeastern Australia, respectively. Rainfall anomalies during the seasons of disease outbreaks were over 43% above normal in southern Africa and 148% above normal in southeastern Australia (**Table 1**). These wet conditions resulted in cooler than normal temperatures and higher than normal vegetation growth, creating ideal habitats for the emergence of virus-infected mosquito vectors [11-14]. On the other hand, the first recognized dengue epidemic in East Africa, affecting populations in northeastern Kenya and Somalia, followed a period of severe drought. Elevated temperatures such as those associated with drought are known to shorten the extrinsic incubation period of arboviruses in mosquitoes [15] and may increase the ability of some mosquito species to transmit human pathogens such as West Nile and dengue viruses [16]. In addition, infrequent replenishment of stored water supply around households, expected during periods of drought, has been shown to increase *Aedes aegypti* populations [17], and may have led to recent outbreaks of chikungunya fever in East Africa [18]. Severe drought coupled with higher temperatures increased the proportion of container-breeding dengue virus mosquito vectors in urban settings leading to the dengue outbreak that persists up to the present [18, 19].

Distributions of LST and NDVI during periods of disease outbreaks are shown in **Figure S4**. We identified locations with outbreak concentrations to illustrate the relationship of LST and NDVI for the following locations: **Figure S4** (A, B) Lincoln, Nebraska, USA, West Nile fever, 40.8069N, -96.6817W, (C, D) Bloemfontein, South Africa, RVF, 29.1183S, 26.2249E, and (E, F) Peterborough, Australia, MVE, 32.9733S, 138.8376E. During the outbreak period in the US location, there was a positive (negative) shift in LST (NDVI) from the long-term distribution, associated with severe drought conditions and a high concentration of West Nile fever cases (**Figure S4A, B**). Conversely, there were negative (positive) shifts in LST (NDVI) for areas of Rift Valley fever (**Figure S4C, D**) and Murray Valley encephalitis (**Figure S5E, F**) outbreaks associated with above-normal rainfall, cooler temperatures, and abundant vegetation growth. In all these cases the disease vectors are ectothermic, thus their emergence and population dynamics responded to weather conditions.

The 2012 epidemic of West Nile fever was the largest such outbreak in the US since the introduction of West Nile virus into the country in 1999. It is also important to note that the 2012 outbreak was concentrated in urban and agricultural areas. This is because most of these altered landscapes have abundant domesticated animals and human-tolerant wildlife species that are preferable hosts for prominent West Nile fever mosquito vectors that include *Culex pipiens*, *Cx. quinquefasciatus*, and *Cx. tarsalis* [20, 21]. The spike in human West Nile fever cases in 2012 can in part be associated with extreme drought and anomalously high temperatures (**Figure S2**): there was a positive shift in the June, July, August (JJA) seasonal distribution of temperatures towards higher temperatures (the mean shifted from ~30°C to 33°C; **Figure S4A**). High temperatures are known to increase the efficiency of transmission of West Nile virus by both *Cx. pipiens* and *Cx. tarsalis* [22-24]. In addition, such high temperatures have positive effects on mosquito vector population development and survival, biting rates, and viral replication within these mosquito species [22].

On the other extreme, Rift Valley fever and Murray Valley encephalitis mosquito vectors (*Culex* or *Aedes* species) favour cooler temperatures associated with heavy rainfall periods in the tropics and sub-tropics [25, 26]. As reported by Turell [27], mosquitoes reared at low temperature (19°C) under laboratory conditions were significantly more susceptible to infection with Rift Valley fever virus than those reared at 26°C. Therefore, the negative shift in seasonal temperatures (DJF 2010/11) in South Africa (**Figure S4C**) and Australia (**Figure S4E**), where the mean shifted from ~40°C to 30°C during this epidemic period of Rift Valley fever and Murray Valley encephalitis, respectively, shows that a cooler environment was conducive to increased mosquito populations, virus infection and replication, and virus transmission. Our findings show that extreme seasonal shifts in weather conditions, regardless of direction, favor different vectors and may lead to increased risk of vector-borne disease outbreaks. Such outbreaks will vary globally depending on geographic location and baseline condition of disease endemism and seasonality, and could favor the globalization of pathogens.

**References for Supporting Information.**

1. Townshend JRG, Justice CO (2002) Towards operational monitoring of terrestrial systems by moderate-resolution remote sensing. Remote Sens Env 83: 351-359.
2. Tucker CJ (1979) Red and photographic infrared linear combinations for monitoring vegetation*.* Remote Sens Environ 8: 127-150*.*
3. Huete A, Didan K, Miura T, Rodriguez EP, Gao X, et al. (2002) Overview of the radiometric and biophysical performance of the MODIS vegetation indices. Remote Sens Env 83: 195-213.
4. Karnieli A, Agam N, Pinker RT, Anderson M, Imhoff ML, et al. (2010) Use of NDVI and land surface temperature for drought assessment: merits and limitations. J Climate 23: 618–633.
5. Wan Z, Zhang Y, Zhang Q, Li ZL (2002) Validation of the land-surface temperature products retrieved from Terra Moderate Resolution Imaging Spectroradiometer data. Remote Sens Env 83: 163-180.
6. Anyamba A, Tucker CJ (2005) Analysis of Sahelian vegetation dynamics using NOAA-AVHRR NDVI data from 1981-2003. J Arid Environ 63: 596-614.
7. Porter JR, Semenov MA (2005) Crop responses to climatic variation. Phil Trans R Soc B 360: 2021-2035.
8. Semenov MA, Porter JR (1995) Climatic variability and the modeling of crop yields. Agr Forest Meteorol 73: 265-283.
9. Gage KL, Burkot TR, Eisen RJ, Hayes EB (2008) Climate and vectorborne diseases. Am J Prev Med 35: 436-450.
10. Gubler DJ, Reiter P, Ebi KL, Yap W, Nasci R, et al*.* (2001) Climate variability and change in the United States: potential impacts on vector- and rodent-borne diseases. Environ Health Perspect 109: 223-233.
11. Nicholls N (1986) A method for predicting Murray Valley encephalitis in Southeast Australia using the Southern Oscillation. Aust J Exp Biol Med Sci 64: 587-594.
12. Knox J, Cowan RU, Doyle JS, Ligtermoet MK, Archer JS, et al. (2012) Murray Valley encephalitis: a review of clinical features, diagnosis and treatment. Med J Aust 196: 322-326.
13. Anyamba A, Linthicum KJ, Small JL, Collins KM, Tucker CJ, et al. (2012) Climate teleconnections and recent patterns of human and animal disease outbreaks. PLoS Negl Trop Dis 6: e1465.
14. Grobbelaar AA, Weyer J, Leman PA, Kemp A, Paweska JT, et al. (2011) Molecular epidemiology of Rift Valley fever virus. Emerg Infect Dis 17: 2270-2276.
15. Hardy JL, Houk EJ, Kramer LD, Reeves WC (1983) Intrinsic factors affecting vector competence of mosquitoes for arboviruses. Annu Rev Entomol 28: 229-262.
16. Kay BH, Fanning ID, Mottram P (1989) Rearing temperature influences flavivirus vector competence of mosquitoes. Med Vet Entomol 3: 415-422.
17. Subra R (1983) The regulation of preimaginal populations of *Aedes aegypti* L. (Diptera: Culicidae) on the Kenya coast. I. Preimaginal population dynamics and the role of human behavior. Ann Trop Med Parasitol 77: 195-201.
18. Chretien J-P, Anyamba A, Bedno SA, Breiman RF, Sang R, et al. (2007) Drought-associated chikungunya emergence along coastal East Africa. Am J Trop Med Hyg 76: 405-407.
19. IRIN (2011) Kenya: Medics overwhelmed as dengue fever spreads. IRIN Humanitarian News and Analysis, http://www.irinnews.org/Report/93848/KENYA-Medics-overwhelmed-as-dengue-fever-spreads. Accessed 2013 April 10.
20. Bowden SE, Magori K, Drake JM (2011) Regional differences in the association between land cover and West Nile virus disease incidence in humans in the United States. Am J Trop Med Hyg 84: 234-238.
21. Kilpatrick AM (2011) Globalization, land use, and the invasion of West Nile virus. Science 334: 323-327.
22. Kilpatrick AM, Meola MA, Moudy RM, Kramer LD (2008) Temperature, viral genetics, and the transmission of West Nile virus by *Culex pipiens* mosquitoes. PLoS Pathog 4: e1000092.
23. Johnson BJ, Sukhdeo, MVK (2013) Drought-induced amplification of local and regional West Nile virus infection rates in New Jersey. J Med Entomol 50: 195-204.
24. Moudy RM, Meola MA, Morin LL, Ebel GD, Kramer LD (2007) A newly emergent genotype of West Nile virus is transmitted earlier and more efficiently by *Culex* mosquitoes. Am J Trop Med Hyg 77: 365-370.
25. Linthicum KJ, Davies FG, Kairo A, Bailey CL (1985) Rift Valley fever virus (family Bunyaviridae, genus *Phlebovirus*). Isolations from Diptera collected during an interepizootic period in Kenya. J Hyg 95: 197-207.
26. Van Den Hurk AF, Craig SB, Tulsiani SM, Jansen CC (2010) Emerging tropical diseases in Australia. Part 4. Mosquito-borne diseases. Ann Trop Med Parasit 8: 623-640.
27. Turell MJ (1993) Effect of environmental temperature on the vector competence of *Aedes taeniorhynchus* for Rift Valley fever and Venezuelan equine encephalitis viruses. Am J Trop Med Hyg 49: 672-670.

**Figure Legends:**

**Table S1**. Crop classification data and sources for the regions examined shown in **Figure S1**.

**Figure S1**. Agricultural regions with extreme weather events during 2010-2012 presented in **Figure1**: 1. US (Texas), 2. SW Russia (Volga District), 3. East Africa (Somalia/Kenya), 4. SW Australia (Western Australia), 5. South Africa (Free State/North West), and 6. SE Australia (New South Wales). Areas shown in red represent areas classified as “Agricultural croplands” from the various crop classification data sources given in Table S1.

**Figure S2**. Land surface temperature (LST) and normalized vegetation index (NDVI) anomalies for JJA 2012 for continental US showing the extent of the drought during summer 2012. Most of the country was impacted with seasonal temperature anomalies between 5-20°C and depressed vegetation conditions, with the departure on the order of -40%, destroyed pasture and rangeland, agricultural lands especially corn and soy growing areas in the mid-west, water resources, and caused extensive fires.

**Figure S3**. Land surface temperature (LST) and normalized vegetation index (NDVI) anomalies for JJA 2011 for the Northern US showing cooler than normal seasonal temperatures (5-20°C below normal) and greener than normal vegetation growth (NDVI departures between 10-60% above normal). These conditions were associated with above-normal and widespread rainfall across the northern US especially in the northwest region.

**Figure S4**. Distribution of land surface temperature (LST) and normalized difference vegetation index (NDVI) during periods of disease outbreaks in selected regions: (A, B) Lincoln, Nebraska, USA: West Nile fever, 40.8069N, -96.6817W, (C, D) Bloemfontein, South Africa: Rift Valley Fever, 29.1183S, 26.2249E, and (E, F) Peterborough, Australia: Murray Valley encephalitis, 32.9733S, 138.8376E.

**Table S1.**

| **Region** | **Crop Classification Source** |
| --- | --- |
| US (Texas) | NASS 2010 Cropland Data Layer: http://www.nass.usda.gov/research/Cropland/SARS1a.htm |
| South Africa  (Free State/North West) | 2002 SADC Land cover database:  (cultivated data layer)  http://gsdi.geoportal.csir.co.za/projects |
| East Africa (Somalia/Kenya)  SW Russia (Volga District)  SE Australia (New South Wales)  SW Australia (Western Australia) | GlobCover 2009:  <http://ionia1.esrin.esa.int/> |

**Figure S1.**


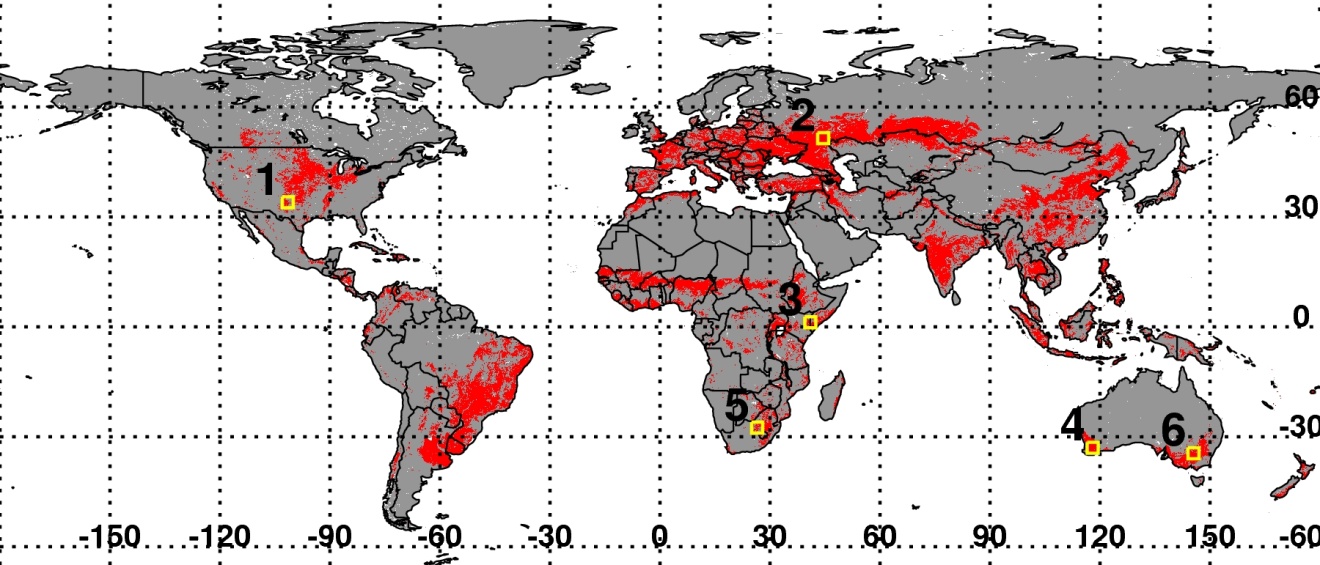


**Figure S2.**

**
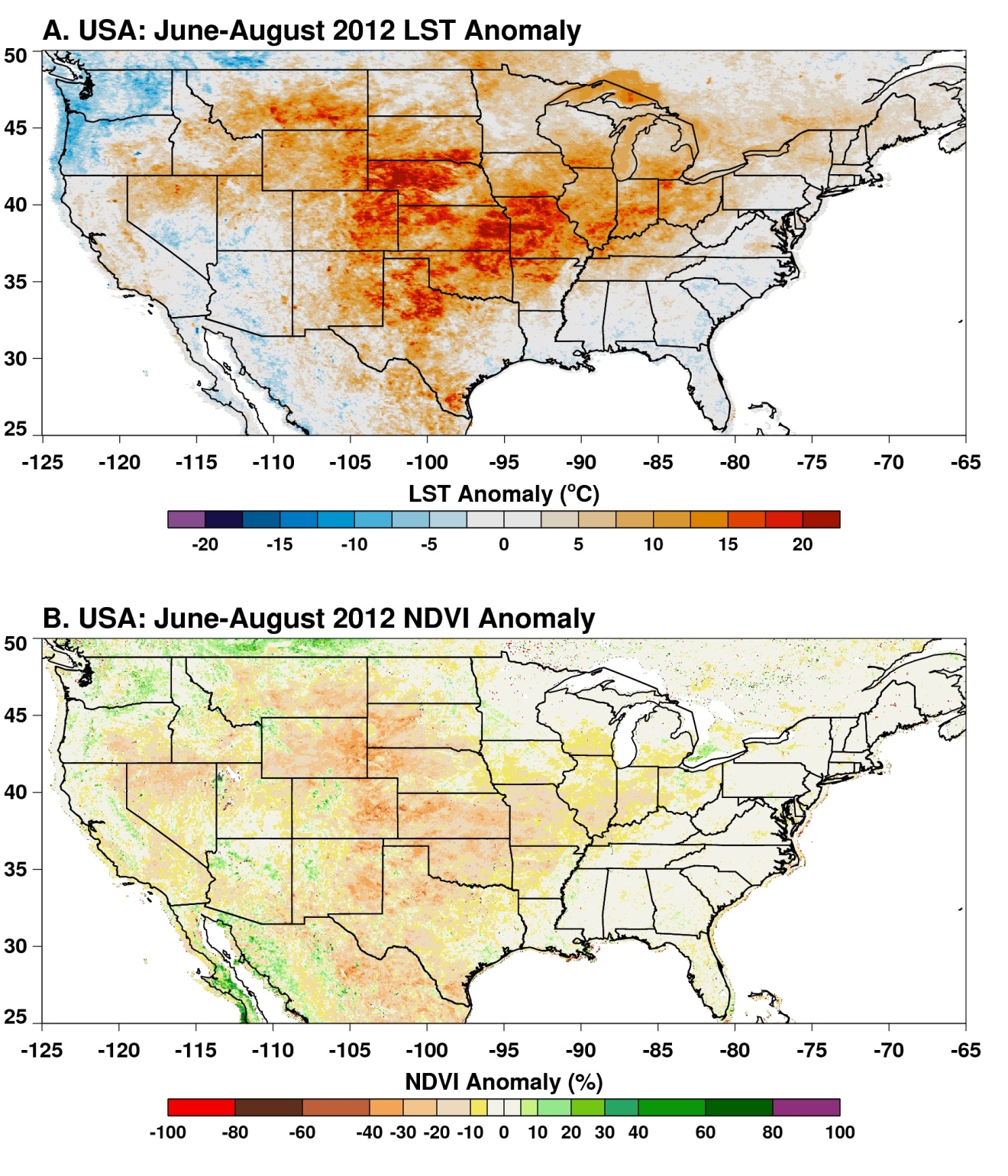
**

**Figure S3.**

**
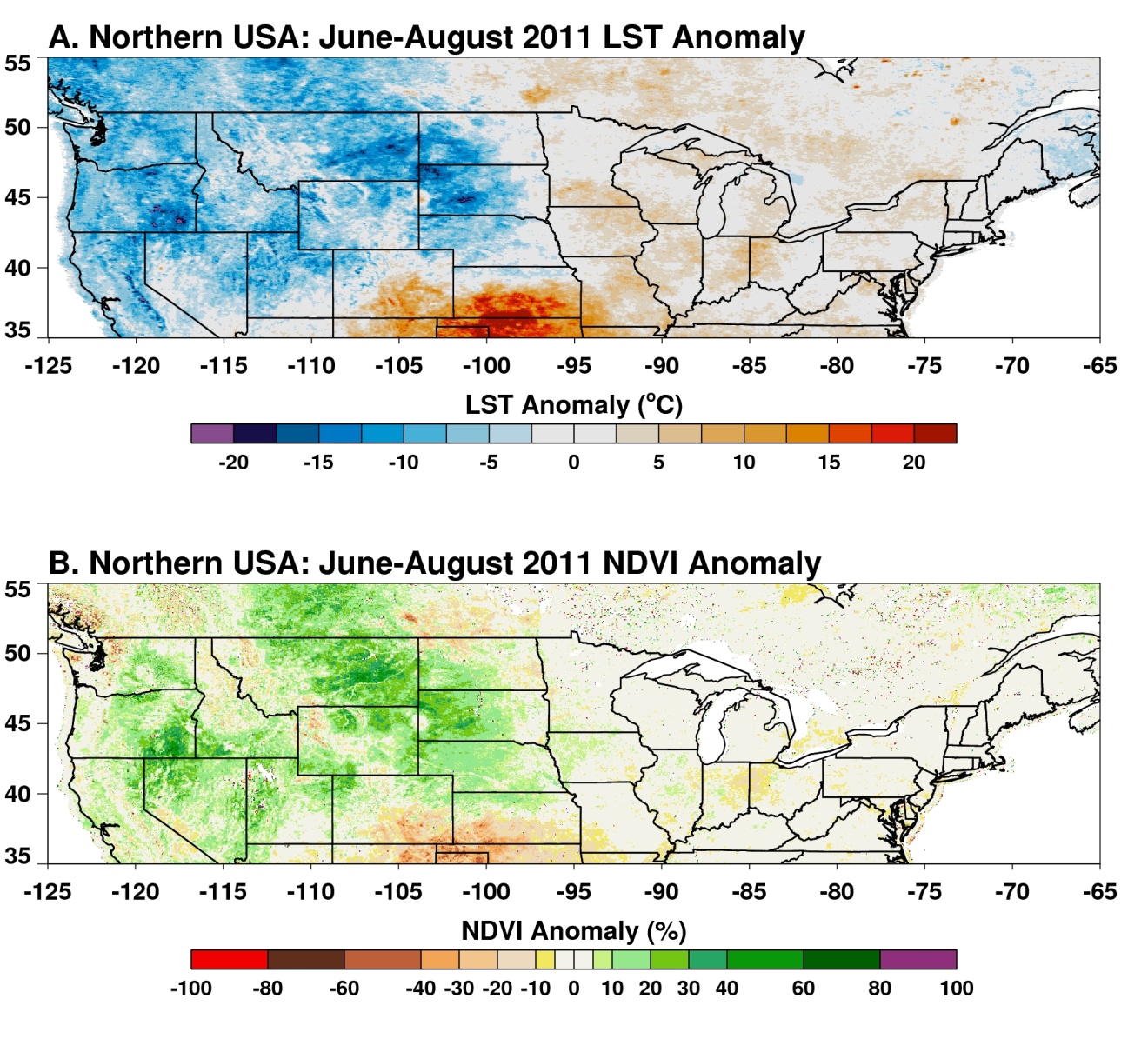
**

**Figure S4.**

**
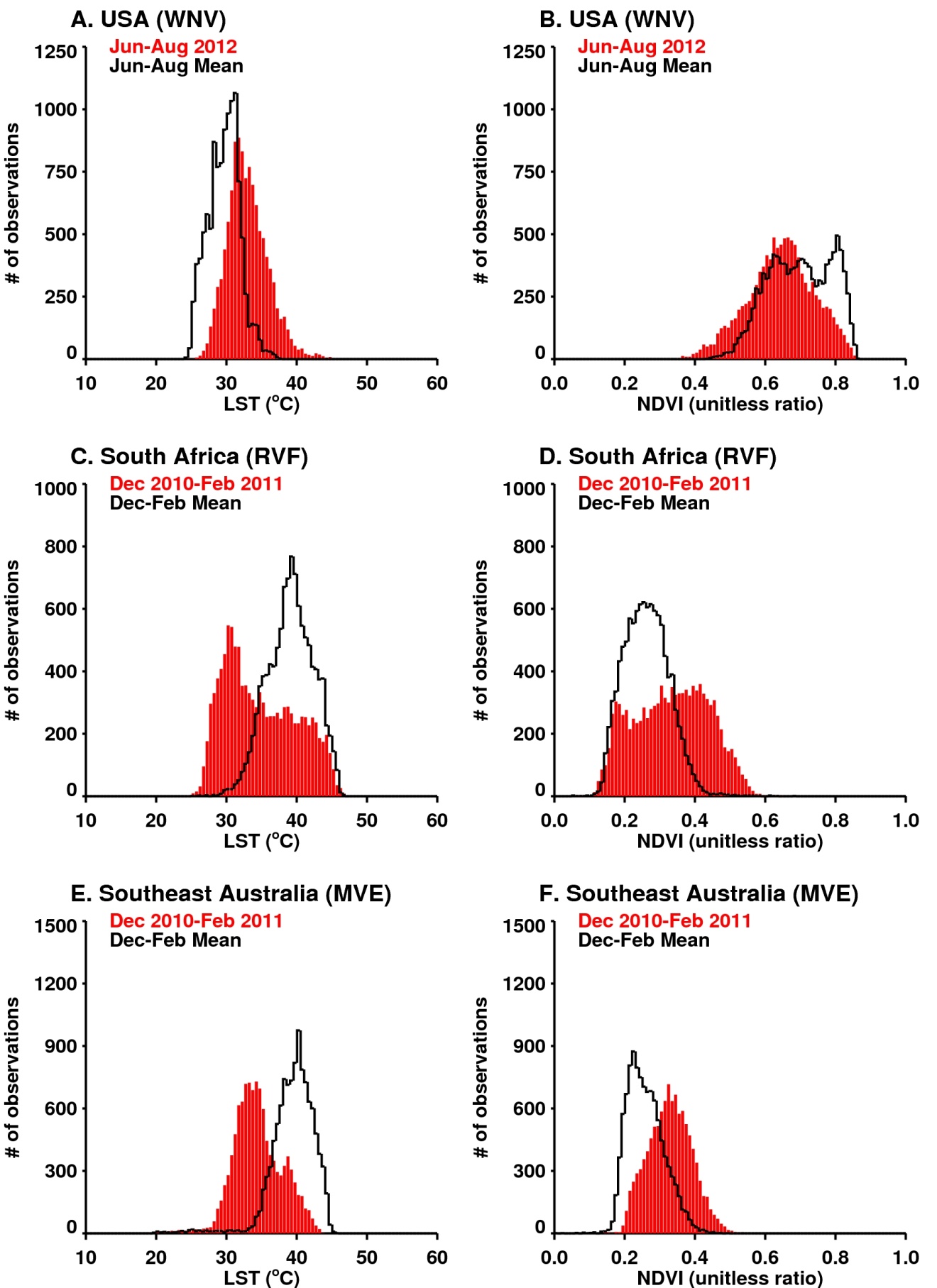
**
